# Factors influencing therapeutic response to pegylated interferon plus ribavirin in the different genotypes of chronic hepatitis C

**Published:** 2014

**Authors:** Mehdi Heidari, Masomeh Bayani, Ali Bijani, Mohammad Reza Hasanjani Roushan

**Affiliations:** 1. Infectious Diseases and Tropical Medicine Research Center, Babol University of Medical Sciences, Babol, Iran.

**Keywords:** Chronic hepatitis C, Treatment, Genotype, Virological response, Sex, IV drug abuser

## Abstract

***Background: ***Different factors like age, sex, route of infection, initial viral load, levels of liver function tests and genotypes may affect response to treatment in patients with chronic hepatitis C. The purpose of this study was to determine the role of these factors in the treatment of these patients.

***Methods:*** From 2004 to 2012, different genotypes of chronic HCV (Hepatitis C virus) patients in Babol, North of Iran who were treated with pegylated interferon plus ribavirin with standard doses (48 weeks for genotype 1, and 24 weeks for genotypes 2 and 3) were entered in the study. HCV RNA was measured during and after treatment based on genotype and protocol. The outcome measure was defined as sustained virological response (SVR) (negative HCV RNA after six months of therapy) was achieved. The data were collected and analyzed.

***Results:*** The mean age of the patients (61 males, 5 females) was 33.82±9.64 years. Twenty seven (40.9%), 37 (56.1%) and 2 (%3) were genotypes 1, 2 and 3, respectively. Twenty one (77.8%) with genotype 1, and 34 (91.9%) with genotype 3 achieved SVR (P=0.045). Fifty-five of 61 men (90.2%) and 2 out of 5 females (40%) achieved SVR (P=0.01). SVR was seen in 22 (88%) of 25 IV drug patients versus in 35 (85.4%) of the non-addict cases (p>0.05). There were no significant differences regarding age, viral load, and liver aminotransferase levels with treatment.

***Conclusion: ***The results show that genotypes 2 and 3, and the male sex had better SVR. Further studies with large number of cases are recommended.

Chronic HCV infection is one of the main etiologic agents causing chronic liver disease in the world. More than 160 million individuals are infected with HCV and most of them are unaware of their infection .The long term effect of this virus in the liver is variable ranging from minimal change to severe fibrosis and cirrhosis with and without hepatocellular carcinoma (1). Without treatment of acute HCV infection, in more than 85% of these cases, chronic infection is developed and these cases comprise more than 25-40% of the total chronic liver diseases in the world (2). Treatment of this chronic infection changed during the last two decades because of the well-recognized pathophysiological effect of this virus in the body, its diagnosis facilities and treatment regimens (1).

At least 6 different genotypes of this virus were diagnosed throughout the world and each one of them has different response to treatment. In the USA, intravenous drug addiction is the main route of this infection and other risk factors of this infection in this country were blood transfusion before 1992, multiple sex partners and dialysis (3). The standard treatment of the disease is administration of pegylated interferon plus ribavirin which changed the efficacy of monotherapy with interferon (10%) to > 70% with combination of ribavirin and pegylated interferon (2). Nowadays, the discovery of the new agents reached the efficacy for treatment to more than 95% (4). Since this disease is common in Iran, and that several factors may influence the outcome of treatment, this study was conducted to evaluate the effect of several different factors in response to treatment.

## Methods

From 2004 to 2012, all cases of chronic HCV infection that referred to the Department of Infectious Diseases in Babol University of Medical Sciences were entered in the study. Inclusion criteria were those who were treated appropriately with different regimens of therapy during this period. The diagnosis of chronic HCV infection was established using PCR in those who were positive anti-HCV test. Genotyping of the positive cases was performed as well. These patients were treated with pegylated interferon plus ribavirin with sufficient doses of therapy considering the genotyping of the virus (2). 

The purpose of this study was to determine the effect of different variables in response to therapy and these issues were age, sex, route of infection, initial viral load, levels of liver function tests and genotypes. During therapy, RVR (rapid virologic response), EVR (early virologic response), DVR (delayed virological response) were checked according to protocol. Viral load was assessed at the end of therapy and 6 months after therapy (2). 

Treatment was defined when sustained virological response (SVR) (negative HCV RNA after six months of therapy) was seen. Failure of therapy was defined when HCV RNA was not reduced to less than two logs (EVR), or HCV RNA was detected at the end of therapy, or the patients who did not achieve SVR. The data were collected and analyzed with SPSS Version 17. T-test and chi-square tests were used to compare the variables when appropriate. Cox logistic regression model was used to estimate the different variables influencing therapeutic response to treatment.

## Results

During this period, 95 cases were assessed, among them 66 subjects completed the treatment regimens and followed up. The mean age of these patients was 33.82±9.64 years (61 males and 5 females). Genotype 1 was seen in 27, genotype 3 was seen in 37 and genotype 2 in 2 cases. The characteristics of the patients regarding viral loads, ALT, AST and other risk factors are shown in table 1. Therapeutic failure was seen in 6 (9%) of patients in genotype 1 and in 3 (8.1%) patients in genotype 3. Among the 27 cases of genotype 1, SVR was seen in 21 (77.8%) of cases versus in 34 out of 37 (91.9%) cases of genotype 3 (95% CI, 1.05-101.23, P=0.045). 

**Table 1:** Characteristics of the patients with chronic hepatitis C and studied variables

**No (%)**	**Variable **
5 (7.6) 61 (92.4)	**Sex** female male
41 (62.1) 25 (37.9)	**Rout of infection** No IV drug abuser IV drug abuser
24 (36) 42 (64)	**Age** **(yr): **
20 (31) 46 (69)	**Base line viral load** .> 400/000 IU/mL ≤ 400/000 IU/mL
9 (14.1) 31 (48.4) 24(37.5)	**ALT IU/L ** 40-200
3 (4.7) 24 (37.5) 37 (57.8)	**AST IU/L ** 40-200
27 (40.9) 2 (3) 37 (56.1)	**Genotypes** 1 2 3

AST, aspartate aminotransferase; ALT, alanine aminotransferase

All two cases with genotype 2 were treated successfully. Among 61 male cases, 55 (90.2%) was treated but among 5 female cases, only 2 (40%) were treated (95% CI, 3.22-5384.97, P=0.01) (table 2). SVR was seen in 22 (88%) of 25 IV drug addict patients versus 35 (85.4%) in non-addict cases (p>0.05). Response Multiple variables to treatment is shown in table 2.

**Table 2:** Estimation of different variables influencing in therapeutic response to peginterferon plus ribavirin in chronic hepatitis C

**P.value**	**OR (95% CI)**	**Failure** **N (%)**	**Treated** **N (%)**	**Outcome**
0.01	131.64 ( 3.22-5384.97)	3 (60) 6 (9.8)	2 (40) 55 (90.2)	**Sex** Female Male
0.206	0.23 (0.02-2.56)	6 (14.6) 22 (88)	35 (85.4) 3 (12)	**Rout of infection ** No IV drug IV drug
0.095	7.70 ( 0.70-84.61)	6 (25) 3 (7.1)	18 (75) 39 (92.9)	**Age** > 35
0.293	0.21 ( 0.01-3.82)	1 (5) 8 (17.4)	19 (95) 38 (82.6)	**Base line viral load ** >400000 IU/ml: ≤400000 IU/ml:
0.091	19.98 (0.62-641.58)	7 (17.5) 1 (4.2)	33 (82.5) 23 (95.8)	**ALT IU/L** > 40 ≤ 40
0.653	0.58 ( 0.06-6.17)	5 (18.5) 3 (8.1)	22 (81.5) 34 (91.9)	**AST** >40: ≤ 40
0.045	10.30 (1.05-101.23)	6 (22.2) 3 (7.7)	21 (77.8) 36 (93.3)	**Genotype** 1 2

AST, aspartate aminotransferase; ALT, alanine aminotransferase

## Discussion

In the past decades, the treatment of chronic hepatitis C has changed dramatically and the discovery of new drugs continues to play a highly significant. The standard therapy that we used in this study included two antiviral agents of interferon or pegylated interferon plus ribavirin. This regimen of therapy is clearly more efficacious than the previous regimens of monotherapy (4, 5). Nowadays, the newer agents like protease inhibitors have been confirmed as treatment of genotype 1. Therefore, the effect of different factors may influence the response of therapy in our region for the selection of therapeutic regimens. In this study, we found no significant effects of response to treatment regarding age, route of involvement, AST and ALT levels at the initiation of treatment and initial viral loads. Our findings are in accordance with the results of Sulkowski et al. in 2008 (6). In their findings, in spite of genotype, high viral loads, advanced liver disease and hemochromatosis and age > 40 years, high BMI and male sex were associated with lower response to therapy. Another study also showed lower response of treatment regarding genotypes 1 and 4, high RNA levels, higher age at the time of infection, male sex and co-infection with HIV or HBV and the daily use of alcohol (7). Other studies also showed lower response rates to treatment regarding cirrhosis, co-infection with HIV, alcohol consumption, and opiate users (8). In this study, we found that response to therapy in genotype 1 was significantly lower than in genotypes 2 and 3. In a study that was performed in 86 cases in South Korea showed that genotype 1 and higher viral loads had lower response rate to treatment regimen (9). In two other studies also showed the lower response rate to treatment compared to genotypes 2 and 3 (10, 11). In our study, we found that the response rate in males was higher than females. Others showed that male sex with higher viral load, higher BMI and fibrosis had lower response rate (12). Another study in 2011 showed that male sex, chronic alcohol user and high BMI, diabetes mellitus and immune defect might associate with higher fibrosis and lower response rate to therapy (13). Other studies also showed lower response rate in genotype 1, higher viral loads, advanced liver fibrosis, and hemochromatosis, age>40 years, steatosis male sex and black race (2, 14, 15). The weakness of this study is the low number of female cases that influenced our analysis and longer than 24 weeks after therapy may be another weakness of this research. The low number of cases in genotype 2 is another weakness of this study. 

In summary, our results indicate that female sex and genotype 1 both are associated with lower response rate.

## Notes

**Funding: **Infectious Diseases and Tropical Medicine Research Center.
